# Atypical cervical cytomorphologic predictors: a descriptive study of pre-cervical cancer patients of low education in Kenya

**DOI:** 10.11604/pamj.2019.33.124.15753

**Published:** 2019-06-18

**Authors:** Esther Muitta, Tom Were, Anthony Kebira Nyamache, Ng’ethe Muhoho

**Affiliations:** 1 Department of Medical Laboratory Sciences, School of Medicine, Mount Kenya University, Thika, Kenya; 2 Department of Medical Laboratory Sciences, School of Public Health, Biomedical Science and Technology, Masinde Muliro University of Science and Technology, Kakamega, Kenya; 3 Department of Microbiology, School of Pure and Applied Sciences, Kenyatta University, Nairobi, Kenya; 4 Department of Pathology, School of Medicine, Kenyatta University, Nairobi, Kenya

**Keywords:** Atypical cells, HR HPV, cervical cancer

## Abstract

**Introduction:**

high risk HPV is the perpetrator of cervical cancer disease, however screening and vaccination is not included in cervical cancer prevention program within public hospitals in Kenya. This descriptive study assessed the association of specific microbial STI and socio-demographic characteristics and practices with cervical cytomorphologic presentations in regards to pre cervical cancer grades amongst health seeking patients attending the reproductive health clinic of Nakuru County referral hospital, a public hospital under newly devolved health services governance.

**Methods:**

a total of 142 patients (AGC/AIS, n=8; HSIL, n=59; LSIL, n=35; controls, n=40) whose median age ranged between 20-70 years were purposively sampled. A structured questionnaire with closed and open ended entries was administered and STI screening including Pap smear examination for cytomorphological profiling done according to revised 2014 Bethesda classification. Associations were established using chi-square and multivariate logistic regression model to determine prediction of cervical atypia manifestations.

**Results:**

a majority of the study participants had only primary education or no education in AGC/AIS (63%) and HSIL (73%) relative to LSIL (49%) and controls (53%) (P=0.017). Koilocyte rates were higher in AGC/AIS (25%), HSIL (52%) and LSIL (77%) compared controls (12.5%) (P<0.0001). ASCUS predominated in HSIL (61%) and LSIL (86%), while almost all AGC/AIS had AGCUS (88%). HR HPV 16/18 infection rates were higher in AGC/AIS (100%), HSIL (80%) and LSIL (83%) relative to controls (10%) (P<0.0001), and was associated with higher risk of having AGC/AIS (OR, 2.0; 95% CI, 1.940-1.947; P<0.0001); HSIL, (OR, 36.3; 95% CI, 9.5-139.5; P<0.0001); and LSIL (OR, 50.1; 95% CI, 12.0-209.0; P<0.0001).

**Conclusion:**

altogether, pre-cervical cancer in Kenyan women is characterized by koilocytosis and ASCUS probably from the high rates of HPV 16/18 infections. Promoting cancer education and screening for high risk HPV infections and pre-cancerous lesions will improve women's reproductive health.

## Introduction

Cervical cancer disease causes reproductive ill health and is among leading global women cancers accounting for at least 68% annual women mortality in the developing world [1, 2]. The disease is preceded by pre cancer status identified by detection of abnormal cells in cervical smears. The Bethesda system (TBS) [3], is employed to classify cells as per their specific atypia such as koilocytosis manifested as Atypia of squamous cells and of undetermined significance (ASC and ASCUS), Atypia of glandular cells and of undetermined significance (AGC and AGCUS), Atypia of squamous or glandular cells of high grade (ASC-H or AGC-H), low and high grade squamous intraepithelial lesion (L-SIL and H-SIL). High risk genotype 16 and 18 human papilloma virus (HR HPV) persistence is implicated in initial atypical changes of cervical epithelia but alone may not be adequate enough to trigger transformation of cells as other determinants have interplay [4-8]. In Kenya, screening for HR HPV subtypes is not included in the cervical cancer prevention policy and neither is vaccination. This study examined HR HPV prediction to cervical cancer development, in addition to other cervical cell atypia manifestation determinants, with the aim of preventing progression of the disease.

## Methods

We assessed the association of HR 16/18 HPV and socio-demographic characteristics and practices with cervical cytomorphologic presentations. This was a descriptive study among women patients attending the reproductive health clinic of Nakuru County referral hospital during the period of January 2014 to December 2014. Ethical approval for this study was obtained from the Kenyatta University Ethics Review Committee (KUERC-KU/R/COMM/51/228) and Nakuru County Referral Hospital Research and Ethics Committee (RII/VOL.I/08) before commencement of research. A total of 142 study subjects were consecutively recruited into the study during reproductive health clinic visits. A structured closed and open ended questionnaire was administered. Questionnaire administration was conducted through a semi interview process as a number of subjects were illiterate and could not interpret the questions on the list. Cervical screening by visual cervical inspection (VIA/VILI test) was conducted. Endocervical scrape smear examination for cyto-morphological profiling was conducted using the revised 2014 Bethesda classification [3] into four pre cervical cancer study groups of: 1) LSIL; n=35; 2) HSIL; n=59; 3) AGC/AIS; n=8 and 4) Control-No evidence of pre cervical cancer; n=40. Those above 20 years and were VIA/VILI positive specifically where sexual debutation had begun were included in the study. Pregnant women were not included in the survey as well as those who were not willing to participate in the study. Blood samples were collected and screened for *Treponema pallidum* and HIV1/2 antibodies [9, 10]. Endocervical specimens were collected for *Neisseria gonorrhea, Chlamydia trachomatis* and HPV antigen detection [11, 12], as well as for scrape smears preparations for Pap smear processing and staining. Stained and mounted smears were microscopically examined for cyto-morphological profiling and categorization using the Bethesda system into distinct pre cancer grades [3]. All Pap smear microscopic examinations were conducted at low power X10 for evaluation of cellular sufficiency and for atypia at high power X40. Smear findings were confirmed by a clinical cytologist. Smear microscopic feature images were captured using the Venus 2.0^®^programme mounted on computer CPU (specific for Venus camera imagery) launched on a Leitz compound microscope. Various photomicrograph images were captured in different pre cancer grades.

**Data management and analysis:** data capture was done using Microsoft office Excel software [13]. After clean up to remove outliers data was exported into SPPS statistical software [14]. Descriptive statistics for frequencies and proportions were generated. Chi-square tests were used to determine associations between dependent variables (pre cervical cancer grades) and independent variables (predictors). Further analysis using multivariate logistic regression models were used to determine the magnitudes of associations between study groups and likelihood tendency (OR) of predicting pre cervical cancer sign manifestation at 95% confidence interval).

**Ethics approval and consent to participate:** ethical approval for this study was obtained from the Kenyatta University Ethics Review Committee (KUERC-KU/R/COMM/51/228) and Nakuru County Referral Hospital Research and Ethics Committee (RII/VOL.I/08). Written informed consent was obtained from all the study participants prior to administering the questionnaire and collection of blood specimens and endocervical swabs and scrapes specimens.

## Results

**Figure 1:**
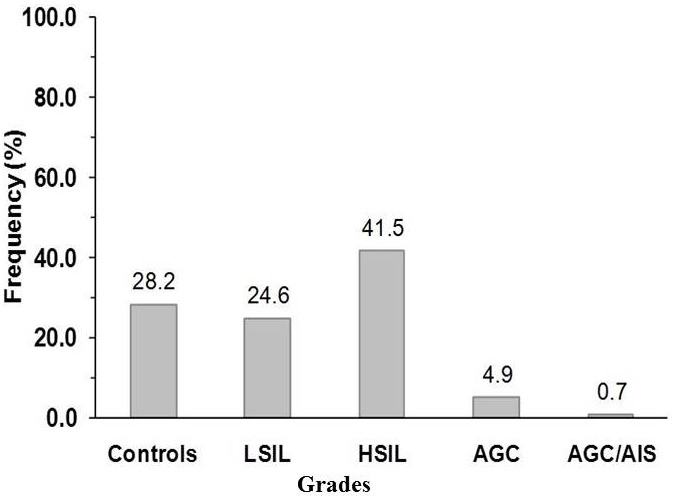
Distribution of pre-cancer grades in enrolled study subjects Enrolled subjects, n= 142. Study groups of controls, n=40 (28.2%); LSIL, n=35 (24.6%); HSIL, n=59 (41.5%); AGC, n=7 (4.9%); and AGC/AIS, n=1 (0.7%). Controls are subjects who did not have evidence of cellular lesion in their smears

**Figure 2:**
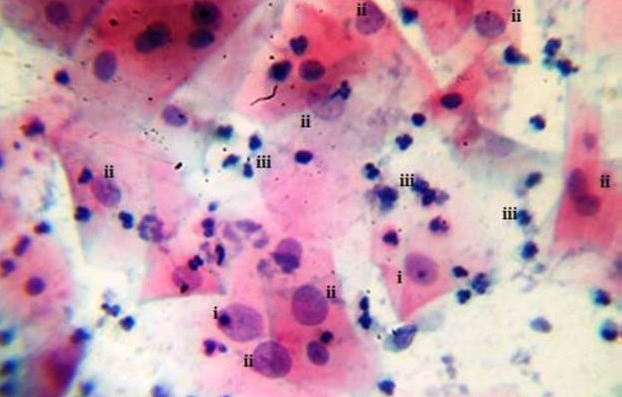
Sampled HSIL study group smear cytomorphology. HSIL smear displaying eosinophillic squames with ASC-H features (i & ii), cells with enlarged nuclear volume and prominent nucleolus (i) without nucleolus (ii) and others displaying prominent coarse chromatin granulation-top right (ii) additionally, presence of polymorphonuclear cells (iii). X40 smear stained using Pap smear staining technique

**Figure 3:**
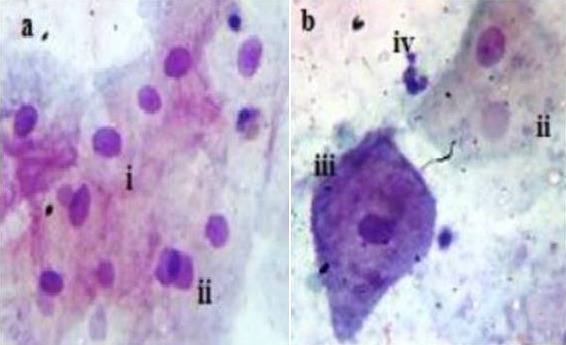
Sampled LSIL study group smear cytomorphology. (a) Basophillic LSIL smear with ASCUS features revealing nuclear membrane irregularity (i), double nuclei (ii). (b) Koilocytic bi-nucleated cell (ii), hyper chromatic cell with peculiar elongated posterior end possibly undergoing metaplasia (iii), polymorphonuclear cell (iv). X40 smear stained using Pap smear staining technique

**Figure 4:**
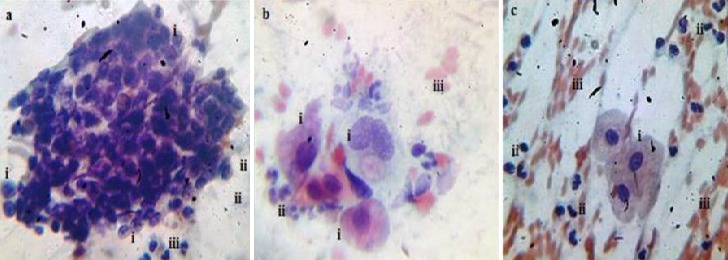
Sampled AGC/AIS study group smear cytomorphology. (a) Endocervical-glandular sheet of cells displaying AGC-H features (i), indistinct hyphae presence (ii) and polymorphonuclear leucocytes (iii). (b) Smear feature of AGC-H (i) interspersed with polymorphonuclear leucocytes and occurrence of indistinct hyphae in the background, (ii). Presence of distorted eosinophilic erythrocytes, (iii). (c) AGCUS cells (i), polymorphonuclear leucocytes, (ii) and sheets of eosinophilic erythrocytic cells, (iii). X40 smear stained in Pap smear staining technique

**Figure 5:**
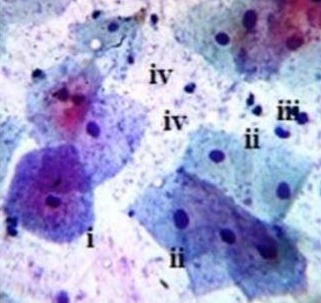
Sampled control study group smear cytomorphology. Polygonal intermediate squames displaying cytoplasmic cytolysis and degradation, (i) and others with intact cell membrane, (ii). Polymorphonuclear cells (iii), doderlein bodies (lactobacillus) suspended in smear content, (iv), X40 smear stained in Pap smear staining technique

The prevalence of cervical atypia from Pap smear test protocol [15] which denoted the grade of pre cervical cancer is shown in Figure 1. HSIL pre cancer grade, (Figure 2) displayed the most abnormal cellular forms examined in smears (41.5%) in comparison to LSIL, 24.6% (Figure 3) and atypia of combined glandular cells of grades AGC and AIS (Figure 4) at 5.6%. Smear examination also revealed that bacterial and fungal infections were present (Figure 4, Figure 5) which caused cervical cell cytoplasmic degradation and cytolysis. The frequency of koilocytes (cells with irregular nuclear membrane, increased N/C ratio, double nuclei with hyperchromasia), Figure 3, was higher in the LSIL study group, 77% in comparison to HSIL 52% and AGC/AIS study group, 25%. At least 12.5% of participants in the control study group smears displayed koilocytic cells. ASCUS presented with high proportions in both LSIL and HSIL study groups, 85.7% and 61% respectively while ASC-H were higher in HSIL Study group (39%) as compared to LSIL (14.3%). In contrast, the frequency of abnormal cells in the AGC/AIS study group was higher in women presenting with AGCUS (87.5%) compared to those of AGC-H (12.5%). Epithelial cellular feature variants associated significantly with pre cancer grades (P<0.0001), koilocytes; χ^2^= 93.952, df, 24; squamous cells; χ^2^ = 193.573, df, 18 and glandular cells; χ^2^ = 139.0, df, 3, (Table 1: data presented are number and frequency (%) of subjects. ^a^This study group were merged subjects presenting with both pre cervical cancer grade AGC and AGC/AIS since only one participant was found to have pre cancer of AIS. ^b^Statistical comparisons for categorical variables were performed using the chi-square test for proportions. ^c^Atypia of squamous cells which was not applicable to study group displaying glandular cell lesions. ^d^Atypia of glandular cells which was not applicable to study group displaying squamous cell lesions. χ^2^, chi-square statistic; df, degrees of freedom). Analysis of smear inclusions revealed higher bacteria, fungi and inflammatory cells presence in AGC/AIC study group (<62.5%) relative to the control, LSIL and HSIL study groups collectively (<58%). One parasite (Trichomonas vaginalis, 2.5%) was detected only in the control group. Corresponding with the presence of microbes, inflammatory cells (polymorphonuclear neutrophils, histiocytes and plasma cells) were present in all the study groups, Table 1. Screening for exposure to selected sexually transmitted immunosuppressive and/or oncogenic organisms, Table 2: data presented are number and frequency (%) of subjects. ^a^This study group were merged subjects presenting with both pre cervical cancer grade AGC and AGC/AIS since only one participant was found to have pre cancer of AIS. ^b^Statistical comparisons for categorical variables were performed using the chi-square test for proportions. χ^2^, chi-square statistic; df, degrees of freedom) indicated that the prevalence of HIV1/2 was higher in women presenting with HSIL grade (25.5%) relative to LSIL and control, 11.4% and 12.5%, respectively (χ^2^= 5.263; df, 3; P=0.154).

**Table 1:** Cytomorphological features examined in study subject cervical smears

Cellular variants	Controls, n=40	LSIL, n=35	HSIL, n=59	^a^AGC/AIS, n=8	c^2^	df	*P*
***Type of cervical epithelia***							
Koilocytes	5 (12.5)	27 (77)	31 (52)	2 (25)	93.952	24	^b^<0.0001
**Squamous cells**							
ASCUS	0 (0)	30 (85.7)	36 (61)	^c^N/A	193.573	18	<0.0001
ASC-H	0 (0)	5 (14.3)	23 (39)	^c^N/A			
**Glandular cells (endocervical and endometrial source)**							
AGCUS	0 (0)	^d^N/A	^d^N/A	7 (87.5)	139.000	3	<0.0001
AGC-H	0 (0)	^d^N/A	^d^N/A	1 (12.5)			
***Inclusions***							
Bacteria (cocci and bacilli)	4 (10)	6 (17.1)	20 (34)	5 (62.5)	73.863	63	0.165
Fungi (hyphae and spores)	5 (12.5)	3 (8.6)	17 (29)	5 (62.5)			
Parasites (trophozoites)	1 (2.5)	0 (0)	0 (0)	0 (0)			
Inflammatory cells (polymorphonuclear histiocytes and plasma cells)	22 (55)	16 (46)	34 (58)	8 (100)			

**Table 2:** STI infections among study subjects

Organism	Controls, n=40	LSIL, n=35	HSIL, n=59	^a^AGC/AIS, n=8	*χ^2^*	df	*P*
HIV1/2	5 (12.5)	4 (11.4)	15 (25.5)	0 (0)	5.263	3	0.154
HR HPV16/18	4 (10)	29 (82.9)	47 (79.7)	8 (100)	62.681	3	^b^<0.0001
*Treponema pallidum*	14 (35)	6 (17.1)	17 (28.8)	3 (20)	3.214	3	0.360
*Neisseriae gonorrhoea*	0 (0)	0 (0)	3 (5.1)	0 (0)	4.158	3	0.245
*Chlamydia trachomatis*	0 (0)	1 (2.9)	4 (6.8)	0 (0)	3.458	3	0.326

Consistent with previous studies showing higher rates of HR HPV16/18 in cervical cancer patients, [5, 16-18] at least three-quarters of the women presenting with pre cancer grades were positive for HPV16/18 serotypes (HSIL, 79.7%; LSIL, 82.9%; AGC/AIS, 100%) compared to controls (10.0%; χ^2^= 62.681; df, 3; P<0.0001). Subsequent regression analysis to determine whether HIV1/2 and HPV16/18 independently predicted pre cancer grades, (Table 3: multivariate logistic regression modeling analyses for HIV1/2 and HR HPV16/18. ^a^HIV1/2 (P value not significant). ^b^HR HPV16/18 (P value significant). Pre cervical cancer grade outcomes of LSIL, HSIL and AGC/AIS were entered as the dependent variable, screening positive for HIV1/2 and HPV 16/18 as the predictor group while screening negative for the detection of both these organisms being entered as the reference. The confounding effect of age, birth control method choice, cervical cancer disease awareness, marital status and education level were controlled for in the regression model. Data are presented as odds ratios (OR) and 95% confidence interval (CI). β coefficient indicates the degree of association differences for model) illustrated that only HPV16/18 was associated with increased risk of having pre cancer grade of AGC/AIS (β, 21.390; OR, 1.947; 95% CI, 1.940-1.947; P<0.0001); HSIL (β, 3.592; OR, 36.323; 95% CI, 9.456-139.525; P<0.0001) and LSIL (β, 3.913; OR, 50.055; 95% CI, 11.993-208.913; P<0.0001. Sero-positivity for syphilis was also detected in all study groups (HSIL, 28.8%; LSIL,^17^.1%; AGC/AIS, 20% and controls,35.0%; χ^2^= 3.214; df, 3; P=0.360). Surface marker antigen positivity for *N. gonorrhea* was only detected in HSIL study group (5.1%) whereas antigen positivity for *C. trachomatiswas* obtained in both HSIL (6.8%); LSIL (2.9%) study groups, Table 3.

**Table 3:** Multivariate logistics model regression analysis of HIV1/2 and HR HPV16/18

Pre cancer grade	β	OR	95% CI	*P*
***HIV1/2***				
LSIL	-0.031	0.969	0.229-4.109	^a^0.966
HSIL	0.993	2.699	0.780-9.342	0.117
AGC/AIS	1.880	6.556	0.241-178.435	0.265
***HR HPV16/18***				
LSIL	3.913	50.055	11.993-208.913	^b^<0.0001
HSIL	3.592	36.323	9.456-139.525	<0.0001
AGC/AIS	21.390	1.947	1.940-1.947	<0.0001

Evaluation of socio-demographic characteristics and practices, Table 4: data presented are number and frequency (%) of subjects or as median (and range) for age (years). ^a^This study group were merged subjects presenting with both pre cervical cancer grade AGC and AGC/AIS since only one participant was found to have pre cancer of AIS. ^b^Analysis of continuous variable of age medians was analyzed using Kruskal-Wallis test. ^c^Statistical comparisons for categorical variables were performed using the chi-square test for proportions. χ^2^, chi-square statistic; df, degrees of freedom) revealed that age medians differed significantly across the study groups (P<0.0001). In between group comparisons showed that women presenting with high grade lesions of both glandular and squamous type were older (AGC/AIS, median, 65.0; range, 50.0-70.0 and HSIL, median, 42.0; range, 27.0-63.0) compared to LSIL and controls (LSIL, median, 38.0; range, 20.0-57.0 and control, median, 34.0; range, 21.0-55.0 years. There was statistical difference in results of education level attained among the study participants (χ^2^=10.226; df, 3; P=0.017) with 73% in HSIL and 63% in AGC/AIS study groups, being women without education or with primary school education levels as compared to LSIL (48.6%) and controls (52.5%). Although marital status did not reveal statistical difference (χ^2^=2.940; df, 3; P=0.401), most of the study participants were married (HSIL, 59%; LSIL, 60.0% and controls, 75.0%). There were no statistical differences in the types of occupations undertaken across the study groups (χ^2^=2.408; df, 6; P=0.879). However, the occupations varied among the study participants, with a majority of the women engaging in informal sector occupations such as farmhands, quarry miners, pastoralists, peasant farmers, or in small businesses (AGC/AIS, 75%; HSIL, 56%; LSIL, 43%; and controls, 53%). There was significant statistical difference obtained on the perception of cervical cancer cause across all the study groups (χ^2^=25.843; df, 9; P=0.002) with varied responses obtained. At least 11% attributed its cause to infections while ~50% responded that they did not know the cause, Table 4.

**Table 4:** Baseline socio-demographic characteristics of study participants

Characteristic	Controls, n=40	LSIL, n=35	HSIL, n=59	^a^AGC/AIS, n=8	c^2^	df	P
Age (years)	34 (21-55)	38 (20-57)	42 (27-63)	65 (50-70)	-	-	^b^<0.0001
**Education**							
≤Primary	21 (52.5)	17 (48.6)	43 (72.9)	5 (62.5)	10.226	3	^c^0.017
≥Secondary	19 (47.5)	18 (51.4)	16 (27.1)	3 (37.5)			
**Marital status**							
Married	30 (75)	21 (60)	35 (59.3)	3 (37.5)	2.940	3	0.401
Single	10 (25)	14 (40)	24 (40.7)	5 (62.5)			
**Birth control use**							
Hormonal	18 (45)	18 (51.4)	33 (56)	0 (0)	21.993	6	0.001
Non Hormonal (devices & barriers)	16 (40)	10 (28.6)	14 (23.7)	0 (0)			
None	6 (15)	7 (20)	12 (20.3)	8 (100)			
Parity							
≥2	22 (55)	19 (54.3)	37 (62.7)	7 (87.5)	1.834	3	0.001
£1	18 (45)	16 (45.7)	22 (37.3)	1 (12.5)			
**Occupation**							
Informal sector	21 (52.5)	15 (42.9)	33 (55.9)	6 (75)	2.408	6	0.879
Small businesses	15 (37.5)	15 (42.9)	19 (32.2)	2 (25)			
Formal employment	4 (10)	5 (14.3)	7 (11.9)	0 (0)			
**Cervical cancer cause**							
Don’t know	20 (50)	24 (68.6)	32 (54.0)	2 (25)	25.843	9	0.002
Infections	7 (17.5)	4 (11.4)	7 (11.9)	0 (0)			
Poor hygiene	4 (10)	7 (20)	18 (30.5)	6 (75)			
Witchcraft	9 (22.5)	0 (0)	2 (3.4)	0 (0)			

## Discussion

HR HPV exposure may not be sufficient enough to cause abnormal transformation of cervical epithelia. Other factors in addition to HR HPV exposure such as having advanced age in women, low immunity, poor chronic ill-health, HIV/AIDS infection, lifestyle and nutritional factors for example organic diet inadequacies, including multiplicity of sexual partners [4, 6, 19-25] are implicated. Pre cervical cancer development has also been linked to under privileged socio-economic state of affairs such as high poverty mainly due to inequality and limitations to access of assets as well as ownership of the same, which berate women in most third world communities such as in Kenya [26 -28]. This study reports cytomorphologic results illustrating a reducing trend in the frequency of koilocytes (~77%, ~52% and ~25% in LSIL, HSIL and AGC/AIS) accompanied by an increasing trend in the frequency of abnormal epithelial cells of high grade cellular lesion types (ASC-H), ~14% in LSIL and ~39% in HSIL, which suggests that higher pre-cancer grade is characterized by manifestation of transformed koilocytes (Figure 2, Figure 3, Figure 4). This pattern confirms what has been obtained from previous studies that have been conducted in Kenya, Europe and America [18, 29-35]. However, in the control group, small proportion of women showed koilocytic cells in their smears (12.5%). This suggests that women is this group are exposed to high risk viral agents that cause cytopathic effects and may potentially develop pre cervical cancer signs should persistence of infection ensue. A study conducted in American women shows similarity in the sense that normal healthy women without cervical epithelial lesions may be candidates of pre cervical cancer development [5].

Moreover, the findings of higher prevalence of abnormal epithelial cells having high grade lesions (HSIL) is attributable to increasing HPV-driven cellular transformation of koilocytic cells to cells of severer levels of abnormality. These assertions are consistent with previous studies showing that persistence of HR HPV especially high risk HPV like serotypes 16 and 18 infection promotes transformation of koilocytic cells to a degree of higher cellular abnormality [5, 36]. Among the immune compromised individuals cell transformation rates could also be high [37]. Therefore, identification of koilocytes in cervical smears is an important indicator of LSIL pre-cancer diagnosis. Viral agents have been linked to cervical cancer [16-18, 34]. While univariate analyses of HIV1/2 infection exposure did not yield statistical difference as displayed in Table 2. HR HPV16/18 significantly associated with LSIL, HSIL and AGC/AIS grades. Further additional multivariate regression modeling analyses confirms the association of HR HPV16/18 with pre-cervical cancer grades Table 3. This implies that the recorded high cases of cervical cancer in pilot studies in Nakuru, [7] are due to high risk HPV strains of 16/18. These analyses are similar with those in cross-sectional hospital based study conducted at Nairobi, Kenya in Kenyatta National hospital showing associations between high risk HPV with HSIL in over 70% of high grade lesions and all squamous cell carcinomas detected in these studies [34]. The sexual risk behaviour of the studied populations in Nakuru could have contributed to high risk acquisition of high risk variant of HPV or some proportions of immunosuppressed individuals and possible low vaccination or screening coverage in Nakuru [5, 38-40].

High median age which was significantly associated with the pre cervical cancer grades (P<0.0001) was encountered, Table 4. This result is in harmony with reports in other studies which imply that having advanced age places a woman at risk of developing pre cancer signs within the cervix as a result of diminishing ovarian hormones due to menopause [29, 30]. In addition, lack of medical check in routine screening of cervical cancer may also contribute to high proportion of median age groups, who may detected this when at advanced stage of cancer. Similarly, knowledge on cause of cervical cancer assessed from varied responses obtained from study participants was linked to pre cancer grades (P= 0.002), as displayed in Table 4. This association could be attributed to low levels of education (P= 0.017) amongst the study participants drawn from the public referral hospital serving Nakuru County residents. Similar studies conducted in other counties in Kenya show consistency in terms of low education levels, illiteracy and ignorance among cervical cancer patients [27, 31]. Eight study participants (5.6%) with a median age of 65 years (range 50-70) years had atypia of glandular cells in which seven had AGCUS and only one was characterized with high grade glandular atypia (AGC-H) from her Pap smear reading. These cells constitute cellular atypia found in the rarer variant of cervical cancer (adenocarcinomatous type). This low rate is consistent with reports from a collaborative survey of 23 different cervical cancer studies globally [38]. These studies indicate low occurrence of glandular atypia in women which could be due to sampling inaccessibility caused by the inversion of the transformation zone (TZ) in cervixes of post-menopausal women hence making it difficult to access during sampling. In advanced age the TZ moves deeper into the endocervical canal [41], away from reach where cellular sampling for pre cervical cancer screening via scrapping, colposcopy or cervicography can be done. This therefore contributed to a less effective detection of this cervical cancer grade type by way of the Pap smear screening test as applied in this study.

## Conclusion

In this study, koilocytosis, ASCUS and HR HPV16/18 infection are predominant features of pre-cancer grades in pre- and post-menopausal women with low education. Eminent manifestation of koilocytosis and cervical cell atypia in all study subject smears signified that productive and ongoing high risk HPV infection was present. HR HPV persistence is paramount to the transformation of cervical epithelia. Unresolved viral infection arises from protracted immunosuppression influenced by indirect aspects ranging from low education including ignorance, illiteracy and poverty, leading to diminished opportunities to health-seek due to the lack of funds for attainment of sustained good nutrition to build a strong protective immunity. Therefore, the government should intensify the roll out of HR HPV screening in the public hospitals as well as enmass vaccination of young women to avert the trigger of HR 16/18 HPV related pre cervical cancer genesis. Success of this program will be an improvement and upscale the cervical cancer prevention policy.

**Limitations:** liquid based cytology (LBC) techniques over the utility of conventional cytology may have reduced microscopic diagnostic overlaps and pitfalls from mimicry with non-diagnostic finding. DNA methods to distinguishing HPV 16 and 18 genotypes as well as Herpes simplex virus (HSV) and Cytomegalo virus (CMV) strains are recommended for incorporation in the STI screen panel.

### What is known about this topic

- Cervical cytomorphologic atypia detection is conducted from Pap smear preparation examinations;
- The Bethesda system is the system used in grading various cellular atypical forms for cervical cancer diagnosis;
- Koilocytosis signifies productive HPV infection.

### What this study adds

- Cytomorphologic feature examination in Pap smears revealed raised koilocytic atypia amongst the study participants;
- Elevated koilocytic atypia detected in examined participant smears signified productive HR HPV infection was present which corresponded with high frequencies of screened HR HPV 16/18 infection amongst the study participants;
- High frequency of participants with low education implies that ignorance, illiteracy and lack of knowledge may lead to diminished opportunities to health-seek early. This may possibly lead to progression of cervical ill health as a result of non-detection of atypical cellular forms and non-screening of persistent HR HPV infections.

## Competing interests

The authors declare no competing interests.

## References

[1] Bray Freddie, Ferlay Jacques, Soerjomataram Isabelle, Siegel Rebecca, Torre Lindsey (2018). Global cancer statistics 2018: GLOBOCAN estimates of incidence and mortality worldwide for 36 cancers in 185 countries. CA Cancer J Clin.

[2] World cancer research fund Cervical cancer statistics.

[3] Nayar Ritu, Wilbur David (2015). The Pap test and Bethesda revision 2014. Cancer Cytopathology.

[4] Parsonnet Julie (1999). Microbes and malignancy: Infection as a cause of human cancer. Ox Uni Press Ldn N Eng J Med.

[5] Burd Eileen (2003). Human papilloma virus and cervical cancer. Clin Microbiol. Rev.

[6] Cancer research UK Cervical cancer risks and causes.

[7] The Kash 7 abstract submission (2017). Trends of leading cancer cases at knh cancer registry.

[8] KNBS KNBS Population and Housing Census results 2009.

[9] HEALGEN Syphilis Antibody rapid detection.

[10] KHB HIV1/2 Antibody colloidal gold rapid cassette.

[11] Liming Bio Gonorrhoea/Chlamydia combo Antigen rapid test kit.

[12] Liming Bio Pre Cervical Cancer HPV16/18 rapid kit.

[13] Microsoft Office version 2010.

[14] IBM (2010). SPSS version 21.0.

[15] Hughes Helena, Dodds Thomas Handbook of diagnostic cytology.

[16] Chua Keng-Ling, Hjerpe Anders (1996). Persistence of human papillomavirus (HPV) infections preceding cervical carcinoma. Cancer.

[17] De Vuyst Hugo, Gichangi Peter, Estambale Benson (2008). Human papillomavirus types in women with invasive cervical carcinoma by HIV status in Kenya. Int J Cancer.

[18] Memiah Peter, Mbuthia Wangechi, Kiiru Grace (2012). Prevalence and risk factors associated with pre-cancerous cervical cancer lesions among HIV infected women in resource limited settings. Aids Res Treat.

[19] Kiviat NB, Paavonen JA, Wølner-Hanssen P, Critchlow CW, Stamm WE, Douglas J (1990). Histopathology of endocervical infection caused by Chlamydia trachomatis, Herpes simplex virus Trichomonas vaginalis and Neisseria gonorrhea. Hum Pathol.

[20] Viiki Merja, Pukkala Eero, Nieminen Pekka (2000). Gynaecological infections as risk determinants of subsequent cervical neoplasia. Acta Oncol.

[21] Anttila T, Saikku P, Koskela P, Bloigu A, Dillner J, Ikäheimo I (2001). Serotypes of chlamydia trachomatis and risk for development of cervical squamous cell carcinoma. Jama.

[22] Samoff E, Koumans EH, Markowitz LE, Sternberg M, Sawyer MK, Swan D (2005). Association of Chlamydia trachomatis with persistence of high risk types of Human Papilloma virus in a cohort of female adolescents. Amer Jour Epidemi.

[23] Del Prado-Lu (2007). Pesticide exposure, risk factors and health problems among cut flower farmers: a cross sectional study. J Occup Med Toxicol.

[24] CDC, STD Trends in the United States (2019).

[25] Lesmes-Fabian Camilo, Binder Claudia (2013). Pesticide flow analysis to assess human exposure in green house flower production in Colombia. Int Jour Environ Res Public Health.

[26] KDHS 2014 Kenya Demographic and Health Survey (KDHS).

[27] Ngugi CW, Boga H, Muigai AW, Wanzala P, Mbithi JN (2012). Factors affecting uptake of cervical cancer early detection measures among women in Thika, Kenya. Health Care Women Int.

[28] Ngugi CW, Boga H, Muigai AW, Wanzala P, Mbithi JN (2015). Barriers to Cervical Cancer Screening in Rural Kenya: perspectives from a Provider Survey. J Community Health.

[29] Vinh-Hung V, Bourgain C, Vlastos G, Cserni G, De Ridder M, Storme G (2007). Prognostic value of histopathology and trends in cervical cancer: a SEER population study. BMC Cancer.

[30] Henley SJ, King JB, German RR, Richardson LC, Plescia M, Centers for Disease Control and Prevention (CDC) (2016). Surveillance of screening detected cancers (colon and rectum, breast and cervix). MMWR Surveill Summ.

[31] Murithi Gatumo, Susan Gacheri, Abdul-Rauf Sayed, Andrew Scheibe (2018). Women's knowledge and attitudes related to cervical cancer and cervical cancer screening in Isiolo and Tharaka Nithi counties, Kenya: a cross-sectional study. BMC cancer.

[32] Solomon Diane, Breen Nancy, Mc Neel Timothy (2007). Cervical cancer screening rates in the United States and the potential impact of implementation of screening guidelines. Cancer J Clin.

[33] Denton KJ, Herbert A, Turnbull LS, Waddell C, Desai MS, Rana DN (2008). The revised BSCC terminology for abnormal cervical cytology. Cytopathology.

[34] Omenge Orang'o, Tao Liu, Astrid Christooffersen-Deb (2017). Use of VIA, Pap smear, or HR-HPV testing in women living with HIV/AIDS for post-treatment cervical cancer screening: same tests, different priorities. AIDS.

[35] Nayar R, Wilbur D (2015). The Bethesda system for reporting cervical cytology.

[36] Eurocytology Cervical cytology Virtual slides.

[37] Massad LS, Ahdieh L, Benning L, Minkoff H, Greenblatt RM, Watts H (2001). Evolution of cervical abnormalities among women with HIV-1-evidence from surveillance cytology in the women's inter-agency HIV. J Acquir Immune Defic Syndr.

[38] Appleby Paul, Beral Valerie, Berrington deGonzales Amy (2006). International Collaboration of Studies of Cervical Cancer, carcinoma of the cervix and tobacco smoking: collaborative reanalysis of individual data on 13,541 women with carcinoma of the cervix and 23,017 women without carcinoma of the cervix from 23 epidemiological studies. Int J Cancer.

[39] Were Edwin, Nyaberi Zablon, Nathan Buziba (2010). Integrating cervical cancer and genital tract infection screening into Mother Child and Family planning clinics in Eldoret Kenya. AfrHealSc.

[40] Blake Kelly, Ottenbacher Allison, Finney-Rutten Lila (2015). Predictors of Human Papillomavirus awareness knowledge: gaps and opportunities for targeted communication strategies. Am J Prev Med.

[41] Hirschowitz Lynn, Nucci Marisa, Zaino Richard (2013). Problematic issues in the staging of endometrial, cervical and vulval carcinomas. Histopathology.

